# Functional Contributions of Antigen Presenting Cells in Chronic Graft-Versus-Host Disease

**DOI:** 10.3389/fimmu.2021.614183

**Published:** 2021-02-24

**Authors:** Chao Hong, Rong Jin, Xiaoqiu Dai, Xiaoming Gao

**Affiliations:** Institutes of Biology and Medical Sciences, Soochow University, Suzhou, China

**Keywords:** chronic graft versus host disease, allogeneic hematopoietic stem cell transplantation, antigen presenting cells, immune tolerance, immune regulation

## Abstract

Chronic graft-versus-host disease (cGVHD) is one of the most common reasons of late non-relapse morbidity and mortality of patients with allogeneic hematopoietic stem cell transplantation (allo-HSCT). While acute GVHD is considered driven by a pathogenic T cell dominant mechanism, the pathogenesis of cGVHD is much complicated and involves participation of a variety of immune cells other than pathogenic T cells. Existing studies have revealed that antigen presenting cells (APCs) play crucial roles in the pathophysiology of cGVHD. APCs could not only present auto- and alloantigens to prime and activate pathogenic T cells, but also directly mediate the pathogenesis of cGVHD *via* multiple mechanisms including infiltration into tissues/organs, production of inflammatory cytokines as well as auto- and alloantibodies. The studies of this field have led to several therapies targeting different APCs with promising results. This review will focus on the important roles of APCs and their contributions in the pathophysiology of cGVHD after allo-HSCT.

## Introduction

Allogeneic hematopoietic stem cell transplantation (allo-HSCT) is a widely used life-saving procedure for patients with hematopoietic malignancies including leukemia, lymphoma as well as other non-malignant diseases related with bone marrow failure. However, its success is markedly compromised by the development of graft-versus-host disease (GVHD) after transplantation due to the histoincompatibility between donors and recipients. Donor alloreactive T cells are first primed through recognition of host alloantigens presented by host antigen presenting cells (APCs), and less often, by donor APCs. Upon preparative conditioning (including high dose chemotherapy and/or total body irradiation) caused gastrointestinal tract or tissue damage, the released pathogen-associated molecular patterns (PAMPs) and damage-associated molecular patterns (DAMPs) stimulate the upregulation of costimulatory molecules and production of inflammatory cytokines expressed in APCs. Such APCs subsequently drive the activation and differentiation of donor alloreactive T cells into effector T cells which contribute to GVHD in target organs (1–3). According to the time of onset and pathological mechanisms, GVHD can be divided into acute GVHD (aGVHD) and chronic GVHD (cGVHD). aGVHD usually starts within the first 100 days after allo-HSCT and is mediated mainly by infused donor alloreactive T cells in the grafts. Accompanied with the process of aGVHD, donor hematopoietic stem cells (HSCs) engraft in host bone marrow and develop into various immune cell lineages. Unfortunately, such donor-derived immune cells could be dysfunctional and autoreactive due to the altered microenvironment unable to support their normal development. Many aGVHD survivors could further develop into subsequent cGVHD which usually begins at a later stage (100 days to 2 years after allo-HSCT), though earlier onset (termed overlap cGVHD when concurrent with aGVHD) is also possible (4, 5).

cGVHD is a life-threatening complication which affects 30%–70% patients who have received allo-HSCT (6–8), with prior episode of aGVHD as the most potent risk factor. It remains a leading cause of late non-relapse morbidity and mortality of patients following allo-HSCT (9). The incidence of cGVHD has been increasing in the past two decades attributed to increased use of old age donors and unrelated/mismatched donors, reduced intensity conditioning regimen and G-CSF mobilized peripheral blood stem cells (G-PBSCs) instead of unmanipulated bone marrow grafts (8, 10, 11). Several curative therapies against aGVHD, such as corticosteroids and calcineurin inhibitors and other immune inhibition drugs, have been successfully developed (12). However, therapies for cGVHD are still challenging due to our poor understanding on its much complex and obscure pathogenesis (13). Conventional treatments for cGVHD are glucocorticoids and immunosuppressive drugs which only achieve disease remission in part of the patients (14, 15). Moreover, systemic glucocorticoids often bring long-term complications which increase morbidity and mortality in patients with cGVHD (12, 16). In recent years, ruxolitinib (a selective JAK1/2 inhibitor) has been used in patients with steroid-refractory cGVHD which showed promising clinical results (17). Other cell based therapy such as extracorporeal photopheresis has also been found to benefit the treatment of cGVHD although the immunological mechanism remains elusive (18).

## Overview of cGVHD in Patients and Mouse Models

GVHD is a complex immunological process involving both innate and adaptive immune responses. cGVHD and aGVHD have distinct pathogenesis albeit they share some common clinical manifestations (19). Unlike aGVHD in which T cells play dominant pathogenic roles (20), the pathogenesis of cGVHD is comprehensive and involves the infiltration of various inflammatory cells as well as the production of auto- and alloantibodies. The complexity of cGVHD immunopathology also indicates a dysfunction of immune tolerance in the hosts after allo-HSCT, which may be part of the reasons for the unresponsiveness of cGVHD patients to the commonly used immunosuppressive agents (21). Tissue and organ damage caused by donor T cell-mediated aGVHD is crucial for initiating cGVHD. Depletion or inhibition of donor T cells in the grafts by anti-lymphocyte antibodies and high-dose cyclophosphamide in the early post-transplantation period could not only prevent aGVHD but also delay the onset of cGVHD (22–25). cGVHD affects not only epithelial tissues (gastrointestinal tract, lung, liver and skin), mostly targeted in aGVHD, but also many other tissues/organs including oral, esophageal, musculoskeletal, fascial, ocular, joint, and even genital tissues (4, 26–29). Attributed to the introduction of National Institute of Health (NIH) consensus criteria, the diagnosis and scoring for cGVHD have been greatly improved in the last two decades. Fibrosis is the most frequently observed characteristic of cGVHD with cutaneous and pulmonary fibrosis (tissue fibrosis manifesting as scleroderma and bronchiolitis obliterans) as the definitive clinical manifestations (4, 30).

Since human cGVHD is very difficult to study mechanistically, various mouse models of cGVHD have been developed in the last decades (31–36). To recapitulate the natural evolution of clinical cGVHD in human allo-HSCT patients, mouse models have been designed with a more precise imitation of clinic procedures including preparative conditioning (total body irradiation), donor and recipient strain combinations (use semiallogeneic F1 mice or minor histoincompatible mice as recipients), and in some models, use of G-CSF-mobilized splenocytes or peripheral blood grafts instead of conventional bone marrow transplantation (BMT) plus purified splenic T cells to induce cGVHD (37, 38). These aspects permit recipients to survive aGVHD and give time for auto- and alloreactive T cells and B cells to develop and cause cGVHD. Inappropriate BMT conditions such as high dose total body irradiation, or high T cell number in grafts, or use of fully MHC-mismatched donors often correlate with an early mortality (within a couple of weeks) after BMT as a result of severe gastrointestinal aGVHD (20, 39). By adjusting to an optimal BMT condition, an autoimmune-mediated pathology could be induced 4-8 weeks after BMT attributable to chronic autoreactive T cell activation and subsequent autoantibody production (40, 41). Considering of the different kinetics with clinical symptoms observed in patients, the disease occurrence in mouse cGVHD models is often absence or only happens at late stage after BMT. In a mouse model of mixed hematopoietic chimerism, the persistence of host B cells and high levels of circulating IgG autoantibodies were found to be associated with the appearance of sclerodermatous cGVHD-like lesions which were observed 7-9 months after BMT (42). In recent years, CD34^+^-stem-cell-humanized NSG mice were found to develop cGVHD late after transplantation (more than 24 weeks). These mice reproduce the full spectrum of pleiotropism of human cGVHD in the absence of prior aGVHD which may serve as a great model for cGVHD related research (43).

In cGVHD, donor T cells developed from engrafted HSCs could be both auto- and alloreactive capable of inducing similar disease when adoptively transferred into secondary allogeneic or syngeneic recipients (44, 45). In these mouse models, pathogenic Th17 cells have been implicated to be causative to cGVHD as well as their roles in aGVHD (46–48). Specific antibody-mediated suppression of IL-17 producing cells reduces histopathological damage of skin, salivary gland and liver in cGVHD (47). In addition, T follicular helper (Tfh) cells play a part in cGVHD as well through interaction with auto- and alloreactive germinal center (GC) B cells *via* expression of both cell surface molecules and IL-21 (41). The pathogenesis of cGVHD is also found to be closely related with deficient development of regulatory cell subsets such as regulatory T cells (Tregs) and regulatory B cells (Bregs) (49, 50). In addition to the contributions of dysfunctional lymphocytes, pathogenic macrophages play important roles in the development of cGVHD, indicating a mutlifactorial pathogenesis of the disease (51, 52). Based on the studies of mouse models, the pathophysiological and immunological evolution of cGVHD should include at least 4 major mechanisms: distorted T cell negative selection in injured host thymus, lack of regulatory cell populations, macrophage-mediated multi-organ fibrosis and loss of B cell tolerance (50–53). cGVHD is a result of immune imbalance between inflammatory immune responses and inhibitory immune mechanisms that maintain immune tolerance. Given that APCs play critical roles in initiation of auto- and alloreactive T cell responses, development/maintenance of central/peripheral immune tolerance, production of profibrotic cytokines as well as auto- and alloantibodies, they are likely important contributors to the development of cGVHD. Below, we review the existing literatures of the functions and contributions of APCs in the pathogenesis of cGVHD (**Table 1**).

**Table 1:** Distinct origins and functions of antigen presenting cells (APCs) in chronic graft-versus-host disease.

Cell type	Origin	Function	Mouse model/Patient
DCs	Donor	Regulate T cell central tolerance (44) May influence T cell peripheral tolerance (54, 55) Impaired cDC expression of MHCII leads to a failure of Treg development (50) GM-CSF induced CD4^+^CD8^-^ DCs promote Treg expansion (56)	(*H2-Ab1^-^/^-^*)B6→C3H (44) Patients (54, 55) B6→B6D2F1 (50) BALB/c→B6 (50) B10.D2→BALB/c (56)
	Host	NA	NA
B cells	Donor	Production of autoantibodies (57, 58) Production of autoanitbodies (59, 60) Promote the expansion of donor autoreactive T cells (61) Interaction with Tfh cells (41, 62, 63) Altered B-cell homeostasis, over-activation of IgG producing B cells, increased numbers of circulating pre-GC B cells and post-GC plasmablast-like cells (64)	DBA/2→BALB/c (57) B6→B10.BR (58) Patients (59, 60) DBA/2→BALB/c (61) B6→B10.BR (41) B6→B6D2F1 (62) Bm12→B6 (62) DBA/2→BALB/c (63) Patients (64)
	Host	Produce autoantibodies in a mixed chimerism mouse model (42)	FVB→BALB/c (42)
Macrophages	Donor	Mediate fibrosis *via* producing of profibrotic TGF-β, induce the differentiation of fibroblasts into collagen-producing myofibroblasts, promote collagen synthesis and deposition (65, 66) Activate and interact with Th17 cells (67) Induce a strong T cell infiltration in the buccal mucosa and labial salivary glands (68) CSF-1 dependent BM derived M2 macrophages induce pathogenesis of cGVHD *via* expression of CD206 and production of TGF-β (51) M2 macrophage over-activation and increased oxidative stress (69)	B6→B10.BR (65) B10.D2→BALB/c (65) B10.D2→BALB/c (66) HSPCs→*hIL-6* Tg NSG* (67) Patients (68) B6→B6D2F1 (51) Patients (69)
	Host	NA	NA
mTECs	Donor	Restore T cell central tolerance and ameliorate cGVHD by adoptive transfer of donor derived TEC progenitors (70)	B6→BALB/c (70)
	Host	Defective T cell negative selection in thymus due to damage of mTECs (71)	B6→BALB.B (71)

*In this study, cord blood-derived human CD34^+^CD38^-^CD45RA^-^ haematopoietic stem/progenitor cells (HSPCs) were transferred into sublethally irradiated hIL-6 transgenic NSG mice.

NA, data not available.

## Dysregulation of Central and Peripheral T Cell Tolerance by Dendritic Cells in cGVHD

Dendritic cells (DCs) at steady state play dual roles in the induction of T cell-mediated adaptive immune response and maintenance of immune tolerance (72, 73). In cGVHD settings after allo-HSCT, DCs are crucial for initiating pathogenic T cell activation in periphery. Their dysfunction also causes failure of autoreactive T cell education in host thymus and loss of T cell peripheral tolerance which contribute to the pathogenesis of cGVHD.

### Preclinical Data

During normal thymopoietic development, autoreactive T cells are depleted in the thymus as a result of negative selection which is mediated by the medullary thymic epithelia cells (mTECs) and the presence of intrathymic autoantigen presenting DCs (73–76). However, in allogeneic BMT scenario, preparative conditioning regimen and donor T cell-mediated aGVHD could damage host thymus and impair thymopoiesis, resulting in dysfunction of negative selection and subsequent release of auto- and alloreactive T cells into periphery (77–79). Allogeneic BMT recipient animals of MHC class II deficient bone marrow grafts developed cGVHD which can be prevented by prior thymectomy (44), indicating a regulatory role of donor DCs in T cell central tolerance during cGVHD. Donor T cells escaped from the thymus of recipient of MHC class II deficient bone marrow grafts are autoreactive and pathogenic owing to the dysfunction of DCs and can cause cGVHD when transferred into secondary recipient mice (44). Interestingly, even host T cells become pathogenic in the absence of DC-mediated central tolerance. Unlike radioresistant tissue-resident macrophages, host DCs are radiosensitive and replaced by donor cells shortly after transplantation. A study reported that host T cells derived from radioresistant intrathymic T cell precursors escaped negative selection in mice lack of host intrathymic DCs and caused dermal fibrosis in mouse cGVHD model (80). After escaping from dysfunctional thymus, auto- and alloreactive T cells further differentiate into effector T cells in periphery. DCs are well known as the most potent professional APCs in eliciting peripheral naïve T cell activation. While host DCs are rapidly eliminated early after allo-HSCT, donor DCs predominate in peripheral tissues and contribute to the development of cGVHD by presenting both host and donor antigens to activate donor T cells *via* indirectly antigen presentation (81, 82).

### Clinical Data

Although the appearance of donor DCs occurs early after allo-HSCT, their reconstitution is impaired and requires a long period of time to complete. Conventional DCs (cDCs) and plasmacytoid DCs (pDCs) are two major DC subsets both of which contribute to the induction of donor T cell tolerance against host organs after allo-HSCT (73, 74, 83). A study of pediatric allo-HSCT revealed that cDC numbers returned to normal level within 300-400 days after transplantation while pDC numbers recovered very slowly in these pediatric patients and were always lower than their age-matched healthy controls up to 7 years after transplantation (54). Another study reported that allo-HSCT patients with sooner or higher pDC recovery profile correlated with improved overall survival, indicating pDC count in peripheral blood of allo-HSCT patients is a significant predictor of long-term outcome after allo-HSCT (55).

### Pathophysiologic Interpretation and Therapeutic Implications

DCs maintain T cell immune tolerance in both thymus and periphery. Peripheral T cell tolerance can be induced *via* direct interaction of inhibitory signaling molecules PD-L1/PD-1 and (CD80/86)/CTLA4 expressed on the surface of DCs and T cells, respectively (84–86). Besides, DCs could also promote donor T cell tolerance *via* expansion of Tregs. In addition to IL-2 dependency, Tregs require costimulatory signals from DCs for their optimal activation and proliferation. Tregs play important roles in the control of pathogenic T cell response and dysfunctional Treg development could cause various autoimmune diseases (87, 88). Decreased numbers of circulating Tregs were found to be correlated with cGVHD in both preclinical and clinical studies (40, 89–91), and adoptive transfer of Tregs could effectively ameliorate cGVHD (92, 93). DCs are important for their role in the induction and maintenance of Tregs and this function is mediated through a MHC class II-dependent interaction (94). It was found that an inflammatory cytokine milieu dominated by TNF during GVHD impairs the MHC class II antigen presentation pathway of cDCs, while MHC class I presentation remains largely intact, and leads to a failure in Treg development which results in a loss of immune tolerance in cGVHD (50, 95). Promoting Treg expansion is a promising approach to prevent cGVHD. Low-dose subcutaneous injection of IL-2 has shown to effectively expand Tregs *in vivo* and ameliorate cGVHD (96–99). A recent study reported that GM-CSF treatment increased CD4^+^CD8^-^ DC number and promoted DC-dependent Treg expansion, thus protected mice against the development of skin cGVHD (56), validating an indirect strategy to prevent cGVHD *via* strengthening DC and Treg interaction.

## Activation and Infiltration of Donor Macrophages Contribute to cGVHD

Macrophages are remarkably plastic innate immune cells which can be found in all tissues and exhibit a vast functional diversity in development, maintenance of microenvironment homeostasis, tissue damage repair as well as innate immunity and adaptive immunity (100–102). Tissue-resident macrophages differ from monocyte-derived macrophages in terms of origin, which has been widely investigated in the last decade as immune sentinels in immune defense and resolution of inflammation (103). They are of embryonic origin and found to reside in majority peripheral tissues and organs, replenished by self-renewal independent of bone marrow monocyte replacement at steady state. However, after allo-HSCT, tissue-resident macrophages can be replaced by donor monocyte-derived macrophages which contribute to the pathogenesis of cGVHD.

### Preclinical Data

In mouse models, accumulating studies support the concept that donor-derived macrophages could facilitate and intensify the pathophysiology of cGVHD (37, 51, 67, 104). It has been revealed that inhibition of donor macrophage infiltration in tissues and organs could ameliorate mouse cGVHD (65). CSF-1 axis controls macrophage development, differentiation and survival and is critical for monocyte-derived macrophage reconstitution after allo-HSCT. In IL-17-dependent cGVHD models of scleroderma and bronchiolitis obliterans, donor bone marrow-derived macrophages were found infiltrating the skin and lung in a CSF-1/CSF-1R-, but not CCL2/CCR2- or GM-CSF/GM-CSFR-, dependent manner and contribute to the pathogenesis of cGVHD. These macrophages express CD206 and TGF-β but not iNOS, identifying them as M2 macrophages (51). Administration of CSF-1R blocking antibodies significantly reduced HSP47^+^ myofibroblasts in the skin, indicating a macrophage-dependent accumulation of myofibroblasts in cGVHD (66). The origin of macrophages is important for their profibrotic gene expression as evidenced by a finding that monocyte-derived alveolar macrophages differ significantly from tissue-resident alveolar macrophages and drive lung fibrosis after BMT (105).

### Clinical Data

In allo-HSCT scenario, host derived tissue-resident macrophages are eliminated and replaced by donor monocyte differentiated tissue resident macrophages with M2 phenotype which are found associated with the development of cGVHD. CD163, a scavenger receptor with immunoregulatory properties, is expressed mainly on M2 macrophages. Examination of biopsy specimens from patients with skin GVHD showed that increased infiltration of CD163^+^ M2 macrophages was a significant predictor for refractory GVHD and poor prognosis (106). Soluble CD163 (sCD163) accumulates in the blood of hosts under oxidative stress or severe inflammatory conditions, as a result of direct secretion by activated macrophages or cleavage of membrane-bound CD163 from cell surface by matrix metalloproteinases (107–110). Intriguingly, plasma sCD163 in allo-HSCT patients is a high risk predictor of cGVHD, indicating a role of M2 macrophage activation and oxidative stress in the pathogenesis of cGVHD (69). Macrophage-derived chemokine and CC chemokine receptor 4 were also found to be closely associated with strong T cell infiltration in the buccal mucosa and labial salivary glands in cGVHD patients (68).

### Pathophysiologic Interpretation and Therapeutic Implications

Activated donor-derived macrophages could mediate tissue fibrosis *via* production of profibrotic cytokine TGF-β, which induces the differentiation of fibroblasts into collagen-producing myofibroblasts capable of promoting collagen synthesis and deposition in cGVHD (65, 66, 111, 112). Pirfenidone, approved by U.S. Food and Drug Administration (FDA) for idiopathic pulmonary fibrosis, can also ameliorate cGVHD by inhibiting macrophage infiltration and TGF-β production (65). A recent study found that type 2 cannabinoid receptor expressed on macrophages played a critical role in the regulation of cGVHD and therapeutic targeting of this receptor by agonist showed beneficial effect in a sclerodermatous cGVHD model (113). Additionally, macrophages could contribute to the pathogenesis of cGVHD *via* interaction with T cells. In cGVHD, alloreactive T cells activate and differentiate into Th1/Tc1, Th17/Tc17, and Tfh cell paradigms in the presence of inflammatory cytokines such as IL-6 and IL-12, while Th17/Tc17 cells play a central role in cGVHD pathophysiology (46–48). IL-17 is a key mediator of pathology in cGVHD and it controls the infiltration of F4/80^+^ macrophages into skin which facilitate the development of scleroderma (51). It should be noted that both pathogenic macrophages and T cells share some common cytokine requirement. IL-6 is a multifunctional inflammatory cytokine which can activate macrophages and also drive the differentiation of pathogenic Th17 cells. By using a humanized cGVHD mouse model through engraftment of human hematopoietic stem/progenitor cells into *hIL-6* transgenic recipient mice, Rintaro et al. reported that co-activation of macrophages and T cells were found in lung and liver and contribute to the pathogenesis of cGVHD (67). *IL-6* gene polymorphism is closely associated with the pathogenesis of cGVHD and anti-IL-6R monoclonal antibody (tocilizumab) has been reported to ameliorate cGVHD in some allo-HSCT patients (114, 115).

## Loss of B Cell Tolerance in cGVHD

At steady state, B cells develop in bone marrow and undergo negative selection which leads to a state of B cell central tolerance to avoid production and release of autoreactive B cells into periphery. Loss of B cell tolerance and aberrant activation of peripheral B cells contribute to the development of cGVHD (116–118).

### Preclinical Data

An intact bone marrow microenvironment is critical for normal B cell lymphopoiesis. Osteoblasts, which could form bone marrow stromal niche for HSCs and B cell progenitors, are targeted by donor pathogenic T cells in GVHD (119, 120). Interestingly, protection of osteoblasts from T cell-mediated damage, by a Treg-expanded graft infusion, could maintain the bone marrow niche for early B cell progenitors and increase the number of pro-B, pre-B and immature B cells in bone marrow and ameliorate cGVHD (121). Aberrant B cell negative selection in host bone marrow causes release of auto- and alloreactive B cells into periphery. These B cells migrate into secondary lymphoid organs and encounter auto- and alloantigens, become activated and then differentiate into plasmablasts or memory B cells *via* interaction with Tfh cells. Through their expression of cell surface molecules and IL-21, Tfh cells promote mature B cell proliferation, differentiation and secretion of auto- and alloantibodies in cGVHD (41, 62, 122). Both Tfh cells and GC B cells are involved in cGVHD and their functions are mutually dependent. Depletion of B cells could suppress Tfh cells in addition to GC formation in cGVHD (63). These data indicate that T-B cell interaction is an important contributor to the pathogenesis of cGVHD. Interestingly, it was reported that donor B cells in transplants, activated by donor T cells, are also efficient APCs to augment the initial clonal expansion and survival of donor autoreactive T cells which are capable of mediating autoimmune-like cGVHD (61). Recently, a study by Deng et al. has reported that extrafollicular CD4^+^ T and B cell interactions are more important and sufficient for inducing cGVHD, while GC formation is dispensable (123). They identified PSGL-1^low^CD4^+^ pre-Tfh-like extrafollicular T cells that were critical for the pathogenesis of cGVHD owing to their interaction with B cells, indicating a much complex mechanism of T-B cell interaction in the pathogenesis of cGVHD.

### Clinical Data

It was originally found in a case report that a cGVHD patient who developed refractory immune-mediated thrombocytopenia after allo-HSCT responded to B cell depletion therapy (124). This finding provided evidence of B cell dysfunction in the immunopathology of cGVHD and suggested a potential way of cGVHD prevention by B cell depletion. B cell development deficiency is often observed in cGVHD patients, indicating an aberrant bone marrow microenvironment failed to support normal B cell lymphopoiesis and selection during cGVHD (125, 126). Insufficient B lymphopoiesis causes post-transplantational B cell deficiency with decreased bone marrow B cell precursors which has been reported in both aGVHD and cGVHD patients after allo-HSCT (127, 128). In addition, there is increasing evidence showing that aberrant peripheral B cell expansion is a feature of cGVHD owing to their dysfunctional regulation of activation and proliferation. For instance, B cells from patients with active cGVHD are in a heightened metabolic state and resistant to apoptosis due to deficient expression of proapoptotic molecule Bim (129). B cell activating factor of the tumor necrosis family (BAFF), which is produced by macrophages, monocytes, DCs, T cells and stromal cells, plays important roles in B cell metabolism, survival and maintaining autoreactive B cell clones (130–132). In cGVHD patients, increased BAFF concentrations and higher BAFF/B-cell ratios correlate with increased numbers of circulating pre-GC B cells and post-GC plasmablast-like cells (64). These circulating pathogenic B cells are capable of autoantibody production without requiring additional antigen stimulation. Besides, other molecules regulating B cell activation and proliferation could also contribute to B cell-mediated pathogenesis in cGVHD. Increased NOTCH2 activation was found to be closely related with robust BCR responsiveness to alloantigens in B cells from cGVHD patients and suppression of BCR-NOTCH hyperactivation by all-*trans* retinoic acid could reduce NOTCH2 signaling and prevent B cell proliferation while maintaining functional B cell responses (133).

### Pathophysiologic Interpretation and Therapeutic Implications

Production of multiple auto- and alloantibodies is a hallmark of cGVHD, and a variety of auto- and alloantibodies have been found to be associated with the severity of cGVHD (134–137). In mouse cGVHD models of scleroderma and bronchiolitis obliterans, these auto- and alloantibodies are found not only the outcome of dysfunctional B cell activation during cGVHD, but also could be causative to cGVHD pathogenesis (57, 58). Alloantibodies against H-Y minor histocompatibility antigens are significantly associated with cGVHD and disease remission (59). Autoantibodies against platelet-derived growth factor receptor have been found to play a role in the development of skin and lung fibrosis in cGVHD *via* stimulating type I collagen gene expression through the Ha-Ras-ERK1/2-ROS signaling pathway (60). It has been reported that microRNA-17-92 expression is required for alloantibody production and IgG deposition in the skin in cGVHD (138). A recent study found that checkpoint regulator SLAMF3 could modulate the activation thresholds of B cell subsets and SLAMF3 blockade markedly enhanced autoantibody production in cGVHD, thereby revealing a role of SLAMF3 in the negative regulation of cGVHD *via* preventing the expansion of autoreactive B cells (139). Since aberrant activation of B cells contributes to the pathogenesis of cGVHD, approaches directly targeting the key downstream kinases of B cell activation have been developed for cGVHD treatment with promising results. Ibrutinib was designed as a selective inhibitor of Bruton’s tyrosine kinase (BTK) and became the first FDA-approved drug for the treatment of steroid-refractory cGVHD in 2017 (140). A small molecule inhibitor of Syk has been found effective in the therapy of cGVHD in mouse models (32, 141). Fostamatinib, a Syk inhibitor drug approved by FDA for the treatment of immune thrombocytopenia, is now under clinical evaluation in patients with cGVHD.

## Functions of Other APCs in cGVHD

Among the non-hematopoietic APCs (e.g., epithelial or stromal cells), mTECs play important roles in the induction of T lymphocyte central tolerance and the pathogenesis of cGVHD. Damage of recipient mTECs caused by alloreactive T cells in the donor grafts leads to defective negative selection of donor T cells and release of autoreactive CD4^+^ T cells into periphery which contribute to the development of cGVHD (71, 77, 142). A recent study has found that transplantation of donor-derived TEC progenitors into cGVHD recipients could restore immune tolerance and ameliorate cGVHD (70). In periphery, non-hematopoietic APCs initiate the initial priming of alloreactive T cells independent of hematopoietic APCs while the latter contribute to the intensification of GVHD (143–145), although most of these studies are based on mouse models of aGVHD. Considering the chronic inflammation and continuing existence of alloreactive T cells in cGVHD, detailed investigation on the role of peripheral non-hematopoietic APCs in pathophysiology of cGVHD is merited.

## Concluding Remarks

**Figure 1:**
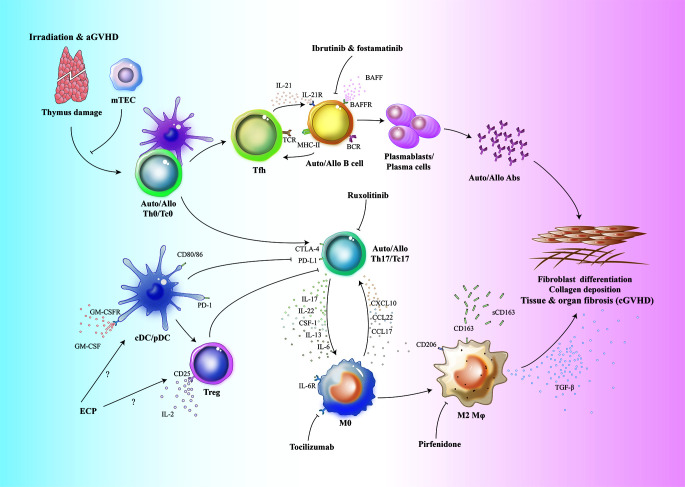
Schematic overview of the functional contributions of APCs to cGVHD. Conditioning regimens such as irradiation, chemotherapy as well as aGVHD cause host thymus damage. Thymic dysfunction contributes to the defective T cell negative selection and release of auto-/alloreactive T cells into periphery. These Th0/Tc0 cells are activated by host or donor DCs and differentiate into auto-/alloreactive Th17/Tc17 and T-follicular helper (Tfh) cells. In germinal center, Tfh cells produce IL-21 which results in activation and expansion of allo-/autoreactive B cells. Elevated levels of BAFF could also contribute to the aberrant B cell expansion. These auto-/alloreactive B cells differentiate into plasmablasts or plasma cells which produce auto-/alloantibodies. Host tissue resident macrophages are eliminated and replaced by donor monocyte derived tissue resident macrophages. These macrophages recruit auto-/alloreactive Th17/Tc17 cells *via* production of chemokines. After migration into target organs, auto-/alloreactive Th17/Tc17 cells further secrete IL-17 to induce more macrophage infiltration. Under the influence of multiple cytokines such as CSF-1, IL-13 and IL-6, donor monocyte derived macrophages are polarized into TGF-β-producing M2 macrophages. The profibrotic cytokine TGF-β, together with auto-/alloantibodies, contribute to the pathogenesis of cGVHD *via* inducing fibroblast differentiation into myofibroblasts which promote collagen synthesis and deposition in target organs and tissues. ECP, extracorporeal photopheresis; Fostamatinib, a Syk inhibitor; Ibrutinib, Bruton’s tyrosine kinase inhibitor; Pirfenidone, an anti-fibrotic drug; Ruxolitinib, a selective JAK1/2 inhibitor; Tocilizumab, anti-IL-6R monoclonal antibody.

While traditional treatments of cGVHD with corticosteroids and other immune suppressive agents are facing more and more challenges, it is of great interest to discover key cellular targets to interfere the pathogenesis of cGVHD. Detailed investigation on APCs in the pathophysiology of cGVHD will provide insights into new potential therapeutic treatments, especially for patients with steroid-refractory cGVHD. Attributed to the broad investigations based on mouse cGVHD models, the functional contributions of different APCs to the pathogenesis of cGVHD have been uncovered which were considered to be promising targets for cGVHD treatment (**Figure 1**). These findings in mouse cGVHD models have been translated into the development of clinical medicines some of which have already showed beneficial results in clinical trials to treat patients with cGVHD (32, 65, 140, 141). However, challenges still remain due to the differences of pathogenesis and kinetics of disease occurrence between mouse models and patients with cGVHD. In addition, there is still lack of effective guidance for selection of optimal therapies for individual patients and none of the drugs available in clinic is effective for all patients with cGVHD. Considering the complexity of cGVHD pathophysiology, comprehensive strategies aiming at multiple APC targets may prove to be more promising in the future.

## Author Contributions

CH, RJ, XD, and XG collected all the literatures for reviewing and wrote the paper. All authors contributed to the article and approved the submitted version.

## Funding

This work was supported by grant from National Key Research and Development Program of China (2017YFA0104502).

## Conflict of Interest

The authors declare that the research was conducted in the absence of any commercial or financial relationships that could be construed as a potential conflict of interest.
